# Perceived access to social support during and after TB treatment in Mbeya and Songwe regions, Tanzania: perspectives from TB patients and survivors set against health care providers

**DOI:** 10.3389/frhs.2024.1273739

**Published:** 2024-07-11

**Authors:** Stella P. Kilima, Godfrey M. Mubyazi, Aneesa Moolla, Nyanda E. Ntinginya, Issa Sabi, Simeon P. Mwanyonga, Denise Evans

**Affiliations:** ^1^ Department of Research Publications and Documentation, National Institute for Medical Research (NIMR), Dar es Salaam, Tanzania; ^2^ Health Economics and Epidemiology Research Office, Faculty of Health Sciences, University of the Witwatersrand, Johannesburg, South Africa; ^3^ Department of TB and Emerging Diseases, NIMR, Mbeya Medical Research Centre, Mbeya, Tanzania

**Keywords:** tuberculosis patients, survivors, emotional, social support, stress

## Abstract

**Introduction:**

Pulmonary tuberculosis (PTB) remains a life-threatening disease in Tanzania, with negative physical, financial, economic and psychosocial consequences to individuals and the society. It mainly lowers the quality of life of patients, survivors and their families, especially those in the poorest and socially deprived categories.

**Objectives:**

To report and discuss a qualitative study that assessed the nature of social support desired and received by PTB patients and survivors. Participants were given a chance to share their experiences and their perceptions on whether the social support they desired had an impact on their treatment-seeking behaviour and treatment adherence.

**Methods:**

Face-to-face interviews were conducted with the three aforementioned groups, purposively selected at a TB clinic between October 2020 and March 2021. The questions covered topics related to the types of social support desired and the sources of support during and after treatment, if any. Interviews were concluded until no new information was obtained. Data analysis was facilitated using NVivo 12 software.

**Results:**

Participants pointed out a need for psychosocial, financial, and material support during and after treatment. However, they sometimes miss support from family/household members or the rest of the community. Because of this experience, they lived with difficulties, facing hardships when required to pay out of pocket for transport during the care-seeking. Survivors testified experience of a denial of support by even their close relatives who regarded them as no longer needing it after recovering. Patients and survivors also reported experience of social isolation as they were believed able to transmit PTB infections. Limited psychological support at the contacted TB clinics was another experience reported. TB clinic staff's experiences confirmed almost all the experiences shared by their clients. With limited support, resilience and self-care were identified as key mechanisms for coping.

**Conclusion:**

Complete recovery from PTB is possible, but reverting to a normal life is difficult without social support. Policies and programs need to increase opportunities for social support for TB patients and survivors. Doing so is likely to improve TB-related treatment, care-seeking practices, and adherence.

## Introduction

### Literature overview

The World Health Organization (WHO) has reported that, tuberculosis (TB), a contagious and airborne disease, was found to be the second killer after COVID-19 in 2022, affecting 10.6 million people globally (1). Over 90% of all the recorded TB cases and deaths are recorded each year in low-and-middle-income countries (LMICs), Africa being one of the regions highly affected (2). Pulmonary tuberculosis (PTB), an airborne mycobacterium disease, is found to claim numerous lives across all infected age groups unless there is timely access to appropriate treatment and care (3). In 2019, an estimated 1.4 million people died of TB worldwide (4), the number slightly declined three years later to 1.3 million (5).

PTB commonly affects the lungs and can be fatal if not treated promptly. The standard treatment for TB in Tanzania a is six-month regimen. The first-line regimen is a combination of rifampicin, isoniazid, ethambutol, and pyrazinamide (RHZE) in the first two months (initial phase) and rifampicin and isoniazid in the last four months (continuous phase) (6). During the entire period of treatment, a supporter is assigned to observe the intake of every dose to ensure that patients take the right anti-TB drugs in the correct doses and at the proper intervals (7). This improves treatment adherence and reduces TB infection in close family contacts and the community. It also prevents drug resistance (MDR) to TB. However, the majority of TB patients in LMICs start treatment late, putting respective patients and their family members at risk (8).

Apart from the physical/physiological threats TB poses directly to the patients, it also leads to negative financial and economic consequences to both the patients and their relatives or family members especially those with whom they live in under the same roof. The physiological disorders, coupled with pain and the financial burden of the disease, have far-reaching effects on the overall health and well-being of affected people. The first directly affected are patients and survivors. Besides the direct pain they physically experience during their illness, patients become socially and psychologically affected too (9). If so severe, the pain may drive the sufferer (patient) to think of committing suicide (10). Survivors also face certain psychosocial consequences after treatment and recovery from the disease. For example, evidence continues to reveal that both the patients and the survivors are sometimes suspected of having TB by the rest of the public/community members. They encounter certain social stigma and discrimination against their state of health by the people perceiving them as contagious (11, 12). Such experiences can lead to post-traumatic stress disorder (PTSD), therefore emphasizing the need for receiving or undergoing specific and important interventions to supplement the already conventionally prescribed medicines (9, 13).

Previous studies justify that social support from any reliable and genuine source such as family members (close relatives), friends, colleagues and health care workers (HCWs), for example, TB nurses and physicians, plays a crucial role in increasing a sense of belonging to the respective social group amongst the mentioned ones and to the rest of the community. Their self-esteem or self-confidence remains stable, and their faith or hope in the recipient of such support (13). However, social support in the TB control program within LMICs is found to remain inadequate. There are occasions when both TB patients and survivors miss the support they need from those expected to assist them. The nature of the support needed could be material or non-material. Material support may take the form of either financial or other kinds of resources for direct or indirect consumption (14). Some kind of support may also be given by, or expected from, the healthcare system or the society members around (15). Lack of social support contributes to loss to follow up (LTFU) among some patients who have initiated treatment (16).

Suggestions continue to be made regarding the need for having in place the reliable sources of support for TB patients and survivors (14). Social support networks are highly recommended and have been proven to play an important role (17, 18). For example, one study in China found the TB patients with family support significantly adhered to their treatment schedules compared to those without (19). The respective patients in this study were provided with support in the form of regular medication supervision to ensure that the directly observed therapy/treatment (DOT) algorithm was adhered to. This happened concurrently with helping to maintain a healthy relationship between the respective patients and their supervisors, including those in the families and the communities to which they belonged. Another form of support given includes the individuals concerned finding themselves receiving spiritual advice and emotional encouragement. However, there is a general disappointment experienced by individuals who miss one or more of the mentioned kinds of support—and makes it difficult for individuals to cope with their lives (19, 20).

A strategy to enable TB patients and survivors to cope with persistent health impairments is needed (11). Additionally, systematic studies show that social support serves as a mediator between self-efficacy and various outcomes, including a depression, stigma, and financial burdens (17, 21, 22). The amount of support that an individual receives plays a crucial role in determining their health seeking behaviour, morbidity and mortality outcomes (23–25). These findings imply that health is not only a product of biological determinants. It is also determined and influenced by social and psychological factors (17, 26). The authors summarise the support needed by patients and survivors into four categories, namely: (1) informational (including advice and guidance), (2) emotional (including expressions of care, as encouragement) which strengthen or heighten the patient's self-esteem, (3) companionship, which instils a sense of belonging for individuals within their social network or group they can rely on for specific needs and (4) material or tangible support, which includes financial assistance, and transportation to meet one's necessities (27, 28). In general, it has been noted that, the relationships between social support, experienced stigma, psychological distress, and quality of life (QoL) among TB patients are still insufficiently understood and are therefore need to be further investigated and discussed (22).

### Theoretical framework

The Stress, Social Support, and Buffering Theory developed by Cohen and Wills in 1985 confirms that experience with long-term illness sometimes push patients and their families into regrettable health, financial and economic crises (29). According to this theory, a disease faced by one person in the family may have spill-over consequences to the family at large. That is, patients with severe or long-term disease conditions may alter the social reality of the family, particularly if the treatment demand is found to result conflicts with developmental tasks of a certain life stage. A consistent correlation has been observed between a lack of social support and adverse psychological as well as biological health outcomes. This is often noted mainly when the patient's concerned lack (miss) the support they need for them to adhere to the prescribed formal medications, leading to LTFU (28, 30). Furthermore, a lack of support intensifies the patient's stress, and this adversely affects such individuals' emotional and biological outcomes. This underscores the urgency of providing positive social and emotional support since doing so facilitates a quick recovery (31).

**Figure 1:**
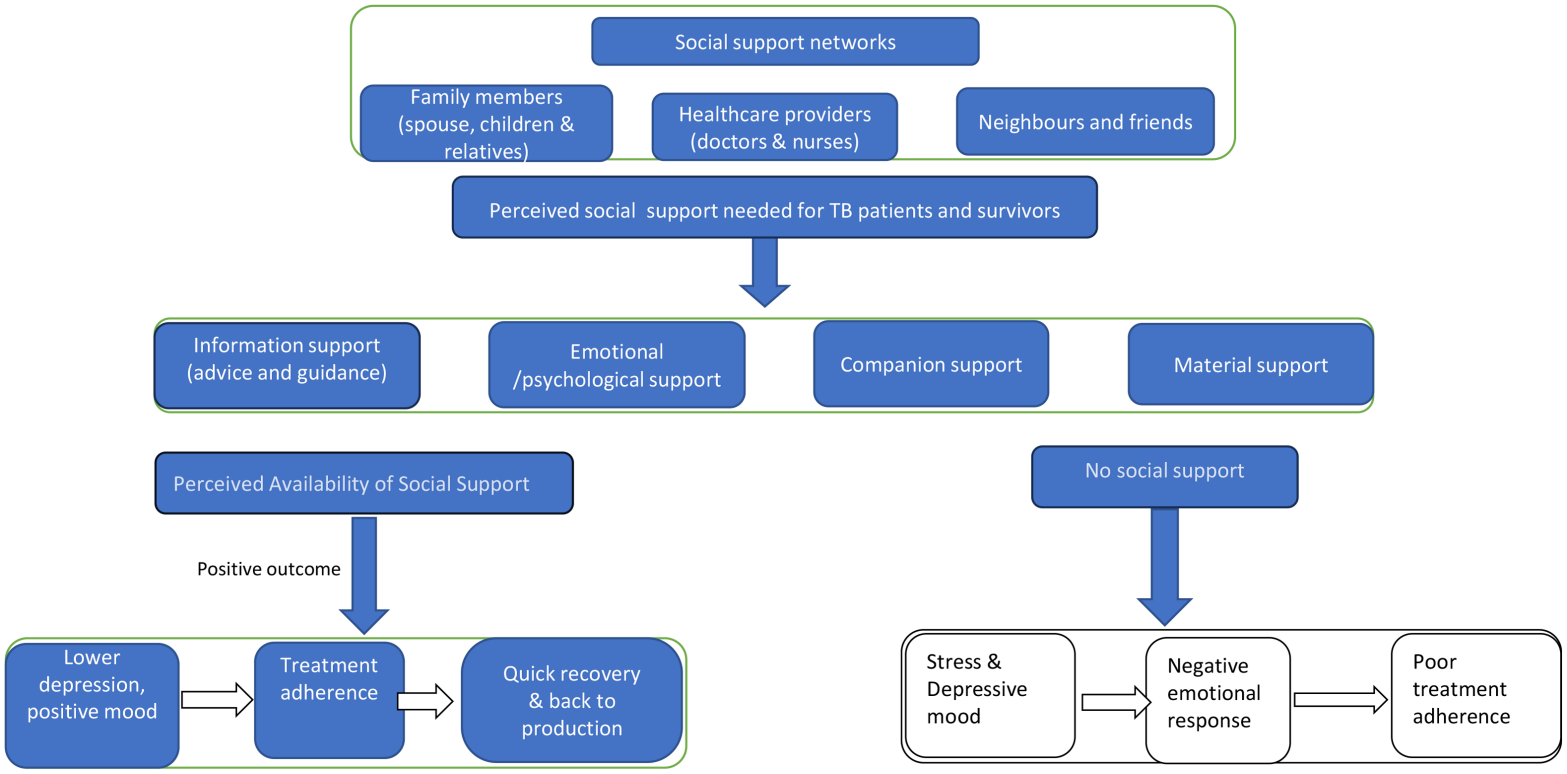
Hypothetical model of the relationship between positive social support and positive outcome.

In summary, Figure 1 describes literature with research-based evidence demonstrating the importance of social support in a TB control program. Figure 1 is also consistent with Cohen and Will (30), who refer to the Stress, Social Support, and Buffering Theory, whose suggestion is that a lack of positive social relationships leads to negative psychological states, such as anxiety and depression. When interpreting this theory in the context of TB patients or survivors, it emphasises the necessity of social support for effective disease control programs or interventions. Such kinds of support are aim at reducing the respective individuals' stressful life conditions and improve their QoL (30). Such a school of thought was the essence of the currently study undertaken to explore the types of social support received as compared to the one perceived as necessary for TB patients and TB survivors during and after their treatment in Mbeya and Songwe Regions, Tanzania.

### Problem statement and rationale for the study

The Global End TB Strategy prioritizes patient-centered care. This means that patients receive respectful and responsive care that considers their individual preferences, needs, and values. All clinical decisions are made with the patient's values in mind. The ultimate goal is to provide high-quality TB diagnosis, treatment, and care to all patients without incurring catastrophic costs (32). The End TB Strategy specifies further that the patient has to be put at the centre of the heart of service delivery whereby he or she feels to be treated/handled with respect, his or her needs and values considered there is room to listen to the individual's questions, concerns and requests (15), offering meals or breakfast or drinks or any other type of nutrition (33), and supporting the individual with a transport facilitation such as fare or fee relief (34). With social and personal circumstances of that person being observed, therefore, not just the immediate requirements of medical equipment (34) should be globally ensured and remain an integral element of the established or the planned TB prevention and treatment programs.

However, countries must have strong TB policies and governance systems to realise practicable translation of the proposed interventions into effective implementation. This also needs to leave room for incorporating insights from researchers whose studies have contributed to generating relevant and field-based policy and program-related evidence. The respective researchers' investigations need to explore the potentially innovative or novel approaches capable of significantly reducing the disease burden and, therefore, improving the QoL of each respective patient and survivor (35). Researchers can also advise the authorities concerned on the key areas needing either new or additional prioritization (36). To encourage the public to value the message of early contact with TB clinics for check-ups and diagnosis, the nature of the service provided at these points of care must meet acceptable quality standards (26). This ensures that individuals who eventually decide to visit the clinic are satisfied with the quality of care received. Another consideration is a need to address the problem of the actual or the possible experience of financial hardships when at the time of service seeking. The standard quality of care/service may include, among other conditions, the type of drugs/medicines prescribed and then used to result into certain treatment outcome(s), and this sometimes depends also on the nature of the process involved in delivering the care received/accessed by the client/patient (37). Part of the process care—that is, the procedure concerned relates to the time spent by the client to wait for service at the clinic/facility, the service providers’ attitude including the language such providers use while interacting with their patients, in addition to the physical environment in which the services are being delivered (38, 39).

As identified above, the quality of care-related factors are found in many countries, contributing to the prevailing dilemma or the stigma attached to deciding when and where to seek care (7, 39). It is further noted that, TB related care does not always stop after the patient completes the prescribed drug course. It is extended to the survivors after treatment (22). Both the conventionally given support such as the one given within the health care facility settings as well as the one obtained from the family, the community around and other sources in the society are important for addressing most of the psychological and the economic challenges individuals who have been traumatised by TB related stress—meaning the patients and survivors, are highly needed for the betterment of the lives of such people (14, 22).

Studies examining at broader determinants of TB, such as poverty, undernutrition, and HIV infection, are essential. They provide valuable insights for policy and program authorities, on potential measures for minimizing the chances of having many TB patients categorized as ‘LTFU, particularly during treatment of drug-resistant TB in adults (14, 34, 40, 41). Despite the already established knowledge on the importance of social support, researchers should continue studying and disseminating findings on the role of social support on TB control policies and interventions/programs in Tanzania (42). Furthermore, the WHO supports research to undertake operational research or implementation science related studies that focus on TB treatment and care. Studies can focus on the practicability (with an emphasis on administration and effectiveness), particularly in TB highly endemic countries. This recognizes that TB control is a multifaceted issue which requires more interventions. Some interventions can be implemented after considering their designs and context-specificity, rather than an overemphasis or over focussing on biomedical determinants and guideline translation approaches (43).

## Materials and methods

### Study design

This study was qualitative. It used cross-sectional survey by design which was guided by descriptive phenomenology, developed by Edmund Husserl (44). This approach enabled the research team to capture key messages from participants' experiences. The study was nested within a large multi-country, multi-center, observational TB cohort study called “TB Sequel” (45).

### Study setting

**Figure 2:**
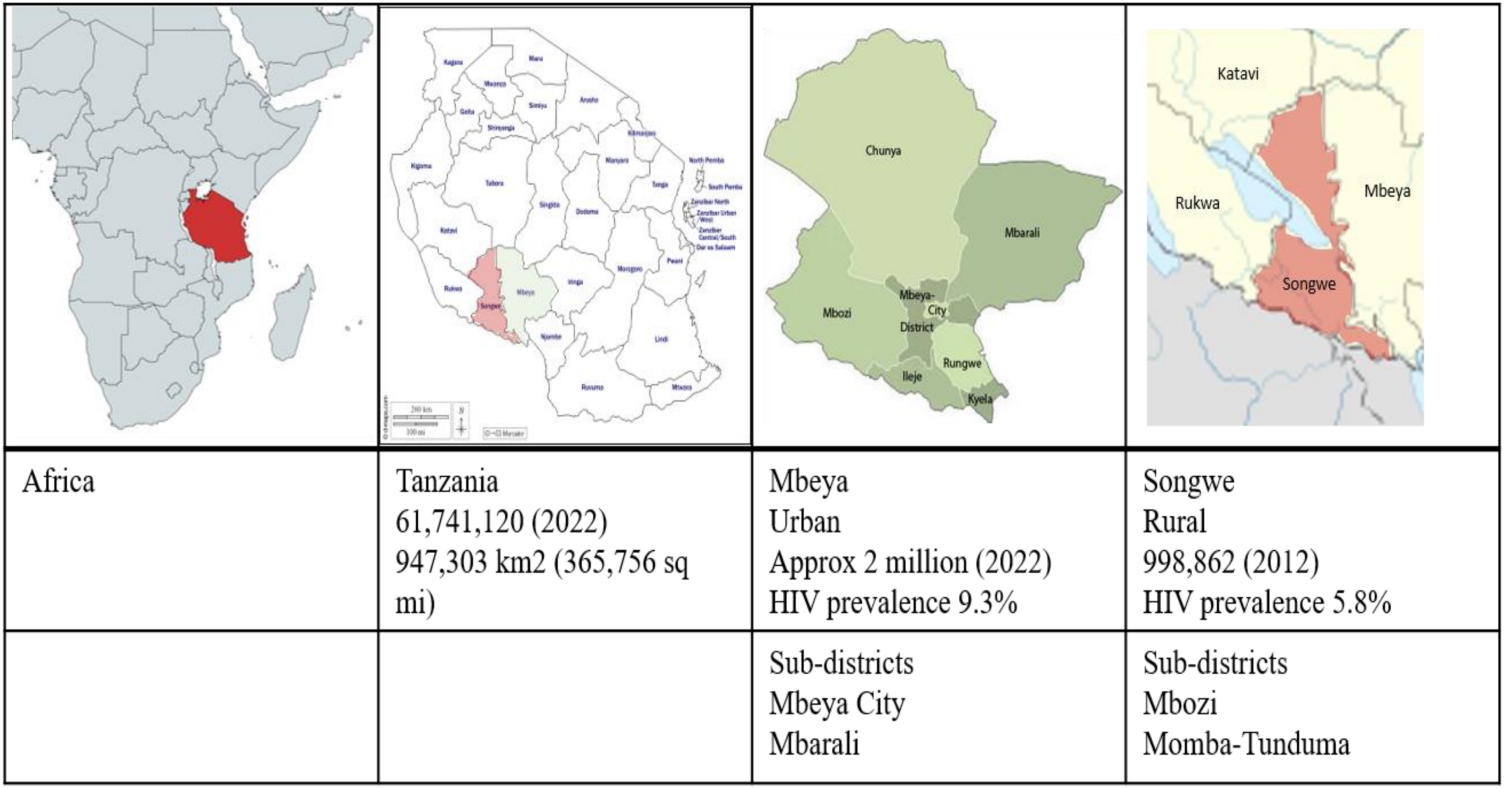
Study setting.

The study was conducted in two regions in the southern highlands of Tanzania, namely; Mbeya and Songwe. The regions were selected due to their high prevalence of TB and HIV at the time of the current study. HIV prevalence was estimated at 9.3% for Mbeya and 5.8% for Songwe (46), both exceeding the national average of 5.0% (47). Two districts were identified in each region, one rural and one being urban. In Songwe, the districts were Mbozi and Momba-Tunduma, while in Mbeya, they were Mbeya City and Mbarali, see Figure 2.

### Study population(s) and their recruitment procedure

The study participants were TB patients, survivors, and healthcare workers (HCWs). Participants were selected purposively, based on their willingness to participate in the interviews on days they visited the TB clinic for the service. Table 1 shows the sizes of the samples for each category of the participants. HCWs included individuals who were service providers for TB patients and TB survivors, for more than six months at the selected TB clinics, within each of the respective regions. All participants were eligible for enrolment in the study after they confirmed their willingness to voluntarily participate by signing the informed consent (IC) form. Measures were taken to ensure that their agreement was given freely without coercion.

**Table 1:** Study population and recruitment criteria.

Target sample size	TB patients (18)	TB survivors (12)	TB HCWs (12)
Eligibility	Adults ≥18 years confirmed TB positive, enrolled in the TB sequel clinic	Adults ≥18 years Who had completed their TB treatment in the last 24 months. Enrolled in TB sequel cohort coming for their scheduled visits. Confirmed TB negative and reside from the four selected districts.	HCWs working in the twelve (12) selected TB clinics. In each clinic, one experienced HCW was selected to participate in the IDI.
Recruitment sites	Being from the selected four districts. Attending TB sequel clinic at Mbeya Zonal Referral Hospital for treatment. Purposively selected	Being from the selected four districts. Attending TB sequel clinic at Mbeya Zonal Referral Hospital for follow-up. Purposively selected	Purposively selected from the twelve (12) clinics selected to refer new TB patients to the TB sequel project.
How identified	Patients attended in the TB clinic from 10/2020–03/2021. The data collection activity conducted during COVID-19 hence contributed to over-estimating stress.	Continued attending the TB sequel clinic from 10/2020–03/2021 for further medical follow-up and additional care, including receiving services intended for the management of possible post-treatment physiological, psychological and other health sequelae as recommended.	HCW worked in a TB clinic for not less than six months. In a place with more than one staff, the most experienced was selected.
How invited to participate	Those who agreed to sign an Informed Consent form, not being critically ill.	Agreed to sign an Informed Consent form, having 24 months or more after treatment completion, confirmed TB negative	Agree to participate and had experience working in the TB clinic

#### Data collection

Before data collection, the interview moderator and note taker attended a three-day training session to familiarize with the study's objectives and methodological approaches. The pretested tools underwent refinement to enhance their user-friendliness, coherence, and increased chances for question understandability and answerability (48). In total, three interview guides were used. Each guide targeted one of the three categories of the respondents. Despite different tools/guides, the issues investigated were similarly, focusing on collecting data that could meet specific study objectives. The questions focused on self-reported TB experience during or after official treatment at the respective clinic. Patients and the survivors had a chance to comment on the types of support, that they received during and after treatment, the importance of social support to them, and comparing this to what they sought and obtained. HCWs commented on the role of social support for TB patients and survivors as well as their role in supporting patients psychologically throughout treatment.

All interviews were conducted in a separate place at a distant location (about 500 m) from the main TB clinic. The distance was to ensure participants’ confidentiality, anonymity, freedom of expression, and confidence. The language used in conducting interviews was Kiswahili, which is the national language spoken fluently in Tanzania. Each interview session lasted between 35 and 45 min. In addition, handwritten notes were taken to supplement audio files (49). The handwritten notes were also validated immediately during the team's daily debriefing session. The audio record and handwritten notes were supplemented by specific annotations for participants’ behaviours, such as indicative crying, sorrowful expressions, laughing, nodding, forced coughing, puffing, denying through head or shoulder shaking (50).

### Data analysis

The interviews were recorded and transcribed verbatim within the same field working days. Two social scientists were involved in transcribing and translating the interviews into English. Translation was done by two individuals independently, who then compared the data. Subsequently, translated transcripts were imported into NVivo 12 software for data analysis and coding. As commonly done when adopting a Grounded Theory (50), the coded data facilitated a thematic analysis. This approach involved examining both the context in which the data were collected and its contents, aligning with the specific objectives of the study. A detailed analysis was performed by three researchers. This involved several steps, including familiarizing the data by reading the transcripts carefully to identify themes and sub-themes. In the process where there was disagreement in the themes and sub-themes, discussions took place until the coders reached consensus (51, 52). All reflective notes were taken into consideration during the data analysis process.

### Ethics approval

The permission to conduct this study received from The Human Research Ethics Committee of the University of Witwatersrand, South Africa (M200570, 29 May 2020), the Mbeya Medical Research and Ethics Review Committee (16 January 2020), and the National Health Research Ethics Committee of Tanzania (Ref. No. NIMR/HQ/R.8a/Vol. IX/3527).

## Results

### Socio-demographic characteristics of the study participants

Out of 30 interviewed participants, 24 (80%) were males. Additionally, 28 (93%) of all participants were aged between twenty-five and forty-five years. Similarly, 29/30 (96%) of respondents had acquired formal education, although at a primary school level. Additionally, 61% (11/18) of TB patients were in the intensive treatment phase (0–3 months) (Table 2).

**Table 2:** Socio-demographic characteristics of participants TB patients and survivors.

Characteristics	Respondent category	
	Patients	Survivors
Region		
Mbeya	9	6
Songwe	9	6
Gender		
Male	14	10
Female	4	2
Age		
25–45	17	11
46–60	1	1
Education		
Primary	14	12
Secondary	3	0
No education	1	0
Marital status		
Married	10	9
Single	3	3
Divorced	5	0
Treatment status		
Intensive phase (0–3 months)	11	0
Continuous phase (4–6 months)	7	0
Treatment completed (>6)	0	12

The 12 HCWs who participated in this study were offering services to TB patients and survivors. Most HCWs 92% (11/12) were females. All HCWs participants had served in the TB clinics for at least two years. The dominant staff cadres among the participants were medical attendants and nurses (Table 3).

**Table 3:** Characteristics of HCWs interviewed in Mbeya and Songwe regions.

Particular/Characteristics	*N* = 12	Mbeya	Songwe
Gender			
Male	1	1	0
Female	11	5	6
Years of service in TB clinic			
2–5	2	1	1
5–9	5	3	2
10 or above	5	2	3
Occupation			
Medical attendant	9	4	5
Nurses	3	2	1
Medical doctor	0	0	0

### Emotional and psychological support

#### Support from HCW

Emotional and psychological support played a crucial role in helping patients and survivors accept their condition and build self-confidence. Without this support, some individuals found it difficult to accept their condition, due to feelings of disrespect and lack of trust. Patients often felt guilt and worried about infecting their family and community members, leading them to fear being isolated due to the prevailing stigma against TB. Such a feeling or fear prompted the respective individuals even to isolate themselves by keeping a distance from other people as a way of preventing them from possible infection. Self-stigma can be reduced or removed through emotional support provided by HCWs during service provision and by the patients' families and communities. Such support helps patients feel a sense of belonging and respect, reducing feelings of abandonment and loneliness. The following statements from respondents confirm this opinion:

“*You know, if HCW speaks to a patient concerning his condition, the patient will be psychologically stable and able to accept the illness condition faced, and will also reduce stress, and the patient concerned will be able to continue taking medicine comfortably.*” (Patient- Mkoani hospital-Mbeya).

Furthermore, most TB patients confessed to not receiving emotional support from the HCWs. The few who received support, asserted that it did not meet their expectations. However, it was interesting to note that many patients acknowledged the demanding schedule of HCWs who often faced heavy workloads. This sometimes made them fail to provide necessary services, including emotional support for psychological rehabilitation, as described below:

“*Emotional support is very important, especially to us TB patients. However, we cannot blame the healthcare provider. We are many, while they are few. Sometimes, staff does not have enough time to speak to you and give you advice as you expect to hear from a professional*” (TB patients, Mbeya).

Some patients commented on lacking emotional support from the HCWs contrary to their expectations and perceived this as negligence, which contributed to their dissatisfaction:

“*I am telling you, we need emotional support, especially from the HCW. As you know, we always trust them, but to my surprise, I never experienced such service, rather than being told you have TB and need to swallow the medicine for six months*” (52 years male, TB patient, Mbozi district, Songwe).

#### Psychological support from peers or fellow patients

Some patients highlighted the importance of getting psychological support from fellow patients, practice which is often observed when waiting for services at the TB clinic/facility. Through sharing experiences patients encourage each other based on their stories. They teach and remind each other about helpful issues, which improved one another's level of understanding or awareness about the disease, as exemplified below:

“*I came here very sick and down. I thought I was going to die any time. I met one patient who highly encouraged me by telling me that he was in bad condition when he came here compared to me. After taking medicine for one month, he changed completely and recovered, this encouraged me, and I also believe I will healed*” (TB patient, Mbozi district, Songwe).

“*In the beginning, I was very down, thanks to my fellow patients, who encouraged me from their own experience and advised me to adhere to the treatment to recover from this disease. Their encouragement helped revive my hope; now I am okay*” (TB female patient, Mbeya City, Mbeya region).

Although they encountered several psychological difficulties, TB survivors complained that they did not receive psychological support. They believed that after treatment completion one needs emotional support to enable them to cope. One of the survivors reported:

“*If you completed TB drugs, other services are seen as if they are not necessary. For instance, emotional support is important because some of us facing difficulties need it along with psychological support. These are not provided to us*” (TB Survivor, Tunduma Songwe).

#### Psychological support from family members or other relatives

There was a general narrative that patients and survivors lacked psychological support from their relatives and other community members. One of the stated reasons for this limitation of support was noted to be due to some of the patients with either TB alone or a TB and HIV comorbidity failing to disclose their personal information about these medical conditions. The reluctance to disclose their status has been identified as contributing factor to poor adherence to the recommended treatment and care seeking behaviour. Therefore, identifying those in need of support has proven to be challenging, as evidenced by the following testimony, among others:

“*I came from a family of thirty children. It is difficult to tell them about my situation because you do not know the heart of everybody. Sometimes, I need support, but hesitate to ask them for it.*” (TB patients, Mbeya City).

Healthcare workers (HCWs) play a critical role in providing emotional support to tuberculosis (TB) patients and their relatives. This support is crucial for patients to adhere to recommended treatment or prescriptions. However, a significant challenge is that most patients only report to the health facility when they are already seriously ill and depressed. This delay in seeking treatment is considered to be due to hopelessness and fear of possible death or permanent disability. HCWs believe that emotional support can help TB patients adhere to the treatment course and prescriptions, and reduce the possibility of death and multi-drug-resistant TB (MDR-TB).

In healthcare facilities, directly observed therapy (DOT) nurses and trained community health workers (CHWs) are responsible for providing emotional support to patients and their families. However, this is not frequently done due to staff shortages or a backlog of duties on certain working days.

“*In the beginning, you can find a patient is depressed, and we, as HCWs, mostly support them. We can give emotional support to ensure that the patient is in good condition to accept their situation; by adhering to the treatment course as recommended, healing is possible. Although patients may wish a lot from us, it is impossible because of staff shortage at the TB clinic.*” (HCW Songwe).

### Information support and its implications for service utilization

TB patients explained that it was important for their communities to be sufficiently supported with information on TB. This will potentially help alleviate some of the misconceptions about TB and hence contribute to reducing its spread. There would also be a better response and quicker treatment seeking when one is confirmed to be infected by TB. One patient gave the following narrative:

“*Maybe I should ask for information support—if education does not pass yet, this disease will bother us a lot; The drug is again free of charge, but without this education, the disease will not reach an end. So, do help us learn about the effects of TB, how a person should live after getting sick, and so on*”. (TB patient, Mbozi district, Songwe region.

TB survivors also reported that they needed information support, particularly on how to integrate with others within the community after completion of TB treatment as well as to avoid re-infection. They need to be educated since they return to the same environment and people. However, a few participants reported getting information after asking healthcare workers, for example:

“*I asked the doctor to tell me how will I stay in the community and avoid another TB infection because I saw my neighbour who completed TB, but now they confirmed him TB positive; I was afraid it might happen to me, then the doctor educated me, I wish everybody who completed drugs should get such kind of education*” (TB Survivor, Tunduma Songwe region).

HCWs, in collaboration with CHWs emphasised the need for TB education within communities. However, reaching all the villages is difficult due to insufficient budget allocations:

“*We are giving community education to community members in the villagers; we also have motorbikes for picking samples to send to the Health Facilities. I can say it is not possible to visit all the villages as the budget allocated is not sufficient*” (HCW Tunduma, Songwe)

Additionally, HCWs in TB clinic provided information support primarily to TB patients. For TB survivors, it is rare to receive information support due to the belief they are already informed.

“*TB survivors, we believe they are aware of TB, as some of them used as champions to educate other people and if they see a person with symptoms, they advise them to come and test*” (HCW- Tunduma, Songwe)

### Companion support from family members and other relatives

Findings suggests that TB patients who were escorted to the health facility on their first day felt comfortable and valued. Those who did not have company while waiting for service on their first day were unhappy, lonely, felt neglected, and helpless. One respondent from Momba district in Songwe region testified about the difficult moments that she experienced in the first few days of her testing and diagnosis. She claimed that she was alone while being screened and tested positive for TB. Therefore, she felt lonely, neglected and isolated. In connection with this experience, the HCWs expressed that companion support is crucial, especially in the early days after the initiation of treatment. Patients are usually accompanied and supported by their friends or family members who they trust, and who would remind them to take their medicine daily as well as helped them to maintain their hospital visiting schedule. However, HCWs did not seem to believe in TB survivors' need for companionship or support, except when they had specific difficulties.

### Material support accessibility opportunities, convenience, and satisfactoriness

#### Monetary support

TB patients reported needing financial support, mainly for transport costs to visit the healthcare facility. Financial support was primarily sought, expected or received from close relatives, such as a family member. A few patients reported receiving support from their siblings or other family members with whom they share a blood relationship. These were reported in the following participants' narratives:

“*It’s just as I said at the beginning, that I’m dependent, and my brother is helping me by giving me money for transport to and from the facility. He is very supportive to me*” (Patient, Mbozi district, Songwe region).

“*Some of my relatives are in Dar es Salaam, and when they heard of my sickness, they continued sending me money to assist me in meeting my treatment needs. The money I get some amount is being used for transport to and from the facility*”. (Patient, Mbozi district, Songwe).

Another participant who received monetary support from his spouse said,

“*…my wife is very supportive to my side, is the one making sure I do have money to attend the facility.*” (Patient, Vwawa Songwe region)

However, other patients believed that, although financial support is essential for TB patients, it is difficult to access it from family members or friends:

“*I always wish my relative to help me with some money, so that they can help me for transport and other need, but it is difficult for people just to give you their money for free*” (Patient, Mbarali, Mbeya region)

“*Money support is important to TB patients but very difficult to access, and you cannot believe others never came to say sorry even if they see me being seriously sick, thinking that you will ask them for money… I cannot blame them as they have a difficult situation financially*”. (Patient, Mbozi District, Songwe).

TB survivors also reported on the importance of monetary support after treatment completion. They were thankful for the TB Sequel project, which gave them transport allowance whenever they made follow-up visit.

“*To be honest TB Sequel project helped us a lot by giving us money for transport. If you show your receipt, they give you double amounts to cover a return ticket cost—the to and from home. That the only monetary support I ever received*” (TB survivor, Tunduma, Songwe region)

Excluding the assistance obtained from the TB Sequel project, most patients and survivors reported never receiving financial support from friends or relatives, during or after treatment completion. The concern was that it was not possible to receive money from such sources given the difficult economic situation that most families faced. However, they acknowledged the importance of receiving financial support to access basic dietary and other social needs, although it was difficult to access:

“*Getting Monetary support is impossible people do not have money for themselves; will they have money to give you? That is why we rely on borrowing*” (TB Survivor, Mbeya DC, Mbeya).

“*If you are sick, people may feel compassion for your situation and give you some money. But, when you complete medicine, and they see you starting to gain weight, nobody thinks about giving you a single cent again*” (TB Survivor, Mbeya DC, Mbeya).

However, few TB survivors confirmed having received support from their close relatives, as one of the quotes below indicates:

“*Some of my close friends supported me with a small amount of money for transport to and from the facility, and they know I am not well financially.*” (Survivor, Mbeya DC, Mbeya).

Commenting on the importance of monetary support for TB patients, HCWs reported that they occasionally financially assisted the patients. The assisted patients included those who did not have financial means, or those who had travelled long distances but did not have transport fare to go back home. However, it was impossible for them to help every patient due to lack of funds. In some cases, HCWs were influenced by empathy to support starving patients who also lacked transport fare, as described below:

“*Yesterday, one patient came here walking from a very far-located village, and she was complaining due to leg pain and having no single cent. I felt sympathetic to her and decided to give her 3,000 shillings for paying a bodaboda (motorbike) driver on her way back home*” (HCW, Songwe).

“*It became a time you feel compassion and decide to help the patient with some money. Some of the patients are very poor, so due to lack of money to afford to buy food or pay for transport back home, they decide to take a walk*” (HCW_Mbalizi, Mbeya)

#### Food support

Most patients reported having received food support from family members when they could not participate in productive economic activities, or engage in crop production. TB patients reported to have had easily accessed food support during the crop harvest season since every family in this period had plenty of food in stock.

“*I received food support from my family members; they assisted me getting food because I had nothing to eat; hence I need to ask them for assistance*” (TB Patient, Momba-Tunduma, Songwe).

“*During harvest season, my relative assisted me and my family in getting food; they brought me maize, potatoes, beans, and groundnuts. Until now, I am using the food received from them, and I appreciate their support and pray for them*” (TB Patient, Mbarali-Mbeya)

Findings suggest that an alternative or additional support was received from neighbours. The neighbours brought food or other support in form of gifts, such as cash, that was given to patients or caregivers. Such helpers extend their support out of recognition of their humanity and the burden carried by the caregivers who were staying with the patient for extended period of time, either at home or at the clinic/hospital during severe illness. The following is a statement representing an experience that was expressed by several other participants among the patients:

“*My neighbours came and brought me food such as a box of maize, and others gave me chicken; if you live well with your neighbours and they know you have a sick person in your house, they bring some gift to support you.*” (Female TB relative, Mbalari Mbeya)

Likewise, TB survivors highlighted the necessity of food support for those facing certain TB sequelae that deny them a chance to participate in economically productive activities even after the completion of their treatment. If given food support could enable such individuals to return to their normal health conditions, leading to participation in routine or day-to-day productive activities. However, some of the TB survivors interviewed reported that they had never received food support from any sources, since farming and other economic activities continued as usual, which enabled one to have at least food for subsistence use.

“*Since I started getting well, I never get food support from anyone. I proceeded with my daily activities, and although sometimes I did not have enough food for myself and my family’s needs, I had not sought support from anybody. You know what, they will be surprised that I have recovered yet still bothered them*” (TB survivor, Mbarali Mbeya)

HCWs concurred with patients and survivors that food support is essential to seriously ill patients who cannot afford to buy food. However, they also explained that there was a need for patients and their caretaker, family members, as well as their community to be regularly educated and sensitized on nutritional matters. They included the importance of growing adequate food and keeping reserves in stock as well as following health diets before and after taking the prescribed TB medicines. The reason for this was that patients who take anti-TB pills on an empty stomach complained of side effects, which may contribute to the loss to follow-up (LTFU) in the TB control program. In general, HCWs reported having met or known many patients who were unable to afford or secure food all the time, primarily because they or their families lack enough time to participate in food production or other income-generating activities, as the following statement testifies:

“*Food support is critical to TB patients. The medicine made patients to eating a lot; for those without food, it is difficult to adhere to the treatment*” (HCW,-Mbeya City).

## Discussion

Findings suggest that, personal and family support during chronic debilitating illnesses are prerequisites for patients' and disease survivors’ well-being (53, 54). The findings indicate how those with TB and TB survivors could cope within an unsupported and challenging environment. Patients and survivors tend to expect social support from other people with whom they interact (55–57). Therefore, there is a need to make social support a part of the policies that are created and established programs, to strengthen efforts towards ending TB (38, 53, 54). Findings indicated that there is more to be done with regard to TB treatment than just administering drugs (58). TB patients and survivors need moral support that would make them feel valued while they go through treatment as well as after their illness. This will potentially have a positive effect on their psychological wellness (58, 59).

A patient suffering from TB wishes to see and feel that they are cared for during their times of crisis. This can be shown through empathy, and cooperation or togetherness. In this study, patients appreciated the moral support from their fellow patients because it kept them feeling alive and able to cope with their current situation (60). Additionally, the TB patients and survivors appreciated the support they received from the HCWs'. They suggested that the support is important in helping the patients cope with the disease and adhere to treatment. HCWs' emotional support should be routinely accessible to all patients because this could make them feel better. It will also improve clinical outcomes and increase the patient's compliance with the prescribed treatment (61). Therefore, service providers and the family members or related supporters must ensure that they support the patient with love, empathy, and care (62, 63). Social support is essential in increasing TB treatment uptake and reducing LTFU (28, 64).

Supporting an individual patient or a survivor of a given disease does not necessarily imply giving them money or other material resources, even positive moral attitude in moments of trouble or pain result into significant positive improvement in that person's health and wellbeing (65). Literature shows that when a patient feels respected when they present their illness to a service provider or caretaker, they may be psychologically motivated (65, 66). Some of the individuals who face post-TB lung disease endure lifelong sequelae (65). This condition may traumatize them for many years, especially, in times that they encounter people who mistreat them (38) The feedback received from both categories of participants confirmed that TB is a burdensome disease not only during the moments of physical health deterioration but also after undergoing treatment and getting cured of the disease (38). Therefore, the current study's findings validate the earlier reported evidence justifying the need for a renewed interest and focus on TB's burden and damaging impact, particularly its long-term sequelae on individual patients, their households, and their communities (67).

The present paper also reveals that those who succeed in completing the required treatment, and are eventually clinically cured of the disease may fail to continue contacting the recommended service providers due to financial barriers. For example, survivors may face financial challenges when seeking the recommended healthcare service. They may struggle to afford travel expenses or the cost of foods and drinks while travelling to and from the service provider or while waiting for service at the clinic (16, 68, 69). Reports from patients and survivors indicate this reality and are consistent with the testimonies in previous studies that were conducted in Tanzania, which maintains the worries that the End-TB Strategy's goal may not be attained (70, 71). Such reports also justify the argument made by other viewers that “mitigating the socioeconomic impact of TB is key to the WHO End TB Strategy” (72). Based on all the authors' reports, mainly those cited in the current paper from past studies, it is clear that financial assistance is necessary for TB patients or survivors. The authors point to the need for not generalizing or taking it for granted that the primary TB services, once made available at the designated points of care, are entirely accessed free of user charges as the policy recommends (71, 73, 74). The respective individuals incur some substantial financial/ monetary costs that are not commonly accounted for by the healthcare system or by most analysts because of the hidden nature of such costs (38, 75).

In the health arena, the relationship between social support and illness, on the one hand, and with the mortality outcomes, on the other hand, there are widely documented findings from field studies that were conducted in various countries. Such studies have demonstrated that physical health is not only a well-being outcome or indicator of biological effects of disease such as TB. Social and psychological health also matters in the observed performance of the prescribed medicines or recommended health practice (17, 26, 38). The evidence from similar studies further reveals that material support is not a sufficient motivation enabler or motivator of a person who is facing a given health problem since there are many examples whereby materially wealthy individuals live in states of unhappiness due to illnesses caused by a disease or injury (76).

### Strength and limitations of the study

The present study is based on a comprehensive review of the TB-related literature. It aims to expand the existing knowledge by examining the role of psychological and behavioural factors in influencing individuals' decisions and behaviours related to TB care-seeking. Furthermore, it seeks to address the importance of studying these factors in the prevention and treatment of TB and related infectious diseases, which have significant implications for public health, socio-economic factors, and healthcare systems. The study highlights the crucial role of social support for individuals affected by TB, including those currently battling the disease and those who have recovered but still face financial, moral, or attitudinal challenges that impact their overall well-being and life satisfaction (77, 78). The significance of social support extends to all aspects of life, emphasizing its importance in ensuring the holistic well-being of individuals affected by TB.

The study's specific objectives and the methodology employed in attempting to achieve them were guided by or were conceived in light of, the “Theoretical Framework of the Stress, Social Support, and Buffering Theory”, developed by Cohen and Wills” (30), with hopefully a meaningful and relevant explanation supporting the illustration presented (Figure 1). The additional chapter has been opened since social-behavioural models need more or further exploration for dealing with or addressing at least some if not all of the context-specific challenges, especially at this stage or moment when the Global End TB Strategy needs increased attention towards its ultimate goal attainment. For example, issues relating to social stigma and discrimination against the patients and even the survivors of pulmonary TB reported disappointing the respective disease victims signify the presence of social or customary norms, traditions, beliefs, and practices or behaviours that negatively affect the quality of life of such people as they contribute to one remaining unhappy because of having the disease, besides the physical pain they have faced throughout the illness period. Additionally, the currently reported study observes all the necessary ethical guidelines and procedures for seeking informed consent (IC) and then for the data collection activities to take place as recommended and endorsed by the appropriate research ethics review bodies in Tanzania and South Africa. The team involved in the process ensured that, seriously, the urgency of treating the study participants with respect and causing no harm to those who consented to take part or to keep it as minimum as possible (61, 62) was taken, and this increased the ownership of the study findings reported and the richness or the validity of the evidence gathered and then presented in the current paper.

The respective harm was expected to be caused by the researcher and therefore to be faced by the respondents when such individuals could find themselves being psychologically affected during the interview session because of the nature of the questions or the topics covered. The apology that was made by the interviewer in advance of starting the interview and even at the end of the interview was found to please the respondents whose minds may have been affected when the question posed concerning certain issues relating to some negative recent or long past events that occurred either during or after TB treatment. The cooperation given by the respondents in question in answering the respective questions and by responding to the interview throughout the planned time without any complain, after they were assured of their anonymity and of the confidentiality of any perceived or seemingly sensitive feedback as given by them was a positive indicator of the value given by such people to the study and the trust they had in both the study and the research team. Nevertheless, several limitations were noted. The use of a purposeful sampling strategy partly cannot overshadow the truth about the researcher's bias apart from the bias originating from the study participants' expressed readiness to be interviewed having dictated who could be interviewed. This implies that, possibly, the individuals who were not interviewed since they did not fall into the sample category could, if given a chance, have presented either a largely or a slightly different experience-based views.

Meanwhile, there was a chance that those who were eventually interviewed could not be open for reporting or commenting on everything the interview had covered and instead they remained with certain reservations or decided to keep some facts undisclosed by hesitating that such facts were not to be known by their interviewers. Some of the respondents might have remained with the belief that the interviewer's interest in hearing about were the facts that sounded positive either on their side or the policy and program for which the investigation was being conducted (79). Another obvious shortcoming is specific for the present article/paper that focuses entirely on the qualitative part of the study, with the quantitative one being presented in a separate paper that has been submitted elsewhere. Triangulating these categories of the findings could obviously have increased the strength of the discussions and the concluding remarks about the study, hence making the evidence-based take-home messages targeting policy-makers, programme officers, and a wider audience to feel more informed and comfortable with what has been reported from the study. Therefore, future research works need to ensure the latter shortcoming is either prevented or minimized, particularly for the studies that aim(ed) at employing a mixed-methods design.

## Conclusions and recommendations

The essential nature of having social support along with the medical services for TB delivered through the conventional TB clinics is validated by the findings from current study in Tanzania. This study highlights that, where material kind of support such as those involving a demand for monetary resources is difficult to be provided or accessed, at least moral support could potentially make a difference, particularly to those needing just to note empathy and encouragement from social members around in order to continue living with hope and to reduce their psychological distress experience encounters or incidents. The respective moral support can be offered by, or expected from the frontline HCWs such as those working in the conventional TB clinics and from other community members such as the relatives of the respective support needers' families, peer groups in the society, as well as the public/community in general. However, this is possible or more practicable if all these potential supporting parties are well-sensitized on the negative health implications of stigmatizing and neglecting or isolating the patients and the disease survivors, as discussed above. Meanwhile, a system-wide approach is recommended for dealing with TB-related challenges and overcoming them (38). The policies and interventions that are currently established need to ensure full engagement of all the key stakeholders, amongst whom are the general public members in the national End-TB Strategy. This can be done through frequent reminders around the need for health to checks in an attempt to detect infectious diseases including pulmonary TB, seeking care in timely manner, whilst simultaneously encouraging TB survivors to adhere to the recommended lifestyles and to avoid exposure to new mycobacterium infections. Researchers need to cooperate with the existing policy and program for TB to carry out investigations aimed at monitoring and evaluating (M&E) the status of post-TB illness-related psychosocial traumas such as stress along with a continued disease surveillance systems (38).

Moreover, the extant lessons learned from a number of countries in Africa and beyond indicating how established or introduced programs whereby mechanisms aimed to enable patients and their families to generate a financial income needed to relieve them from burden of the disease are worthy a recognition. Such countries' governments and their allied local and international development partners have, for example, considered a policy framework allowing the establishment of cash transfers in relation to TB control as part of the mechanisms of social support and as mostly advocated to be one of the key poverty reduction strategies. In spite of the so-far observed or recorded success stories from such kind of resource-based support-related cash transfers, the applicability or feasibility the respective transfers have had on one hand and their acceptability in all socio-economic contexts on the other hand are found to need an additional consideration about among other things, more studies or evaluations being undertaken to confirm their potentials or advantages (54, 80).

Thus, in the aim to achieve universal health coverage (UHC), many African countries' governments have through their ministries of Health (MoH) imbibed the advocated idea calling for the establishment safety-net mechanisms to cater for the TB patients and survivors including those that can act as supplements to other strategies available for ensuring translation of the available policy and program opportunities to implement activities with the view to reduce the levels of poverty in their communities (81). Tanzanian is one of the latter countries whose governments allow such an approach, with some demonstrated success stories in areas where they have been tried out through implementation science kind of research projects focusing on how to enable the existing TB program reach a wider targeted beneficiaries especially those seriously needing support for them to access the basic TB-related healthcare services while they are challenged by the income-related poverty states (71, 82).

Furthermore, TB patients and even survivors can live with acceptable levels of resilience through coping if they feel fully receiving empathy and encouragement from service providers, home-based caretakers, and other societal members with whom they live. The encouragement given by the service providers contacted in the TB clinic settings, as well as the moral-based support receivable from their home caregivers and from the rest members of the society/community in which they live puts them in a perspective and a mood of feeling respected and valued. This important psychology-connected element has positive implications for patients or survivors to adhere to the prescribed or recommended medical measures (78). At the same time, it increases the respective support demanders' trust in both the healthcare system, TB-related care seeking, and a hope to live longer, aside the relief they feel to get from the painful moments they have been going through.

Finally, the current paper argues that, both the existing literature and the findings obtained from the currently reported study shed more light on the cruciality of psychological and material support for TB patients and survivors since apart from such people being required to accept the condition, their adherence to the recommended measures and their continuity with their lives as usual are very important if the End TB Strategy were to be boldly evaluated and concluded as having been successful at ensuring the health and wellbeing of the TB affected populations.

## Ethics considerations

Ethical considerations were strictly adhered throughout the study, and as mentioned above, these include seeking of Informed Consent (IC) from every individual participant approached with all the rights of respondents protection observed—confidentiality, anonymity, non-maleficence, information that the key study findings will be dissemination for policy and public domain usage, provision for anyone deciding not to participate or to withdraw from participating in the study even after initially consenting, non-ill persuasion through, for example, use of persuasive and yet coercive or inducive means such as payment. The consenting individuals were asked to signed a separate IC form (83, 84).

## Data Availability

The original contributions presented in the study are included in the article/Supplementary Materials, further inquiries can be directed to the corresponding author.
